# Supplementary material for: Integrative modeling of membrane-associated protein assemblies
Source: Nat Commun. 2020 Dec 4;11:6210. doi: 10.1038/s41467-020-20076-5 (PMC7718903; doi:10.1038/s41467-020-20076-5)
Supplement: Supplementary file 1 — Supplementary Information [file 41467_2020_20076_MOESM1_ESM.pdf]

Supplementary Information

For

Integrative Modeling of Membrane-associated Protein Assemblies

Jorge Roel-Touris[1†], Brian Jiménez-García[1†*] and Alexandre M.J.J. Bonvin[1*]

[1]Bijvoet Centre for Biomolecular Research, Faculty of Science-Chemistry, Utrecht University, Utrecht, The Netherlands.

[†]These authors contributed equally to this work.

[*]To whom correspondence should be addressed. E-mail:

Brian Jiménez-García: b.jimenezgarcia@uu.nl

Alexandre M.J.J. Bonvin: a.m.j.j.bonvin@uu.nl

Fig. S1. Graphical representation of the 1k4c receptor for the different tested scenarios in ZDOCK. The residues masked according to the maximum (ZDOCK-max), average (ZDOCK-avg) or minimum (ZDOCK-min) z-axis coordinate provided by the MemProtDB database are colored in dark gray. The orange regions, thus, represent those residues still allowed to contact with their counterpart.

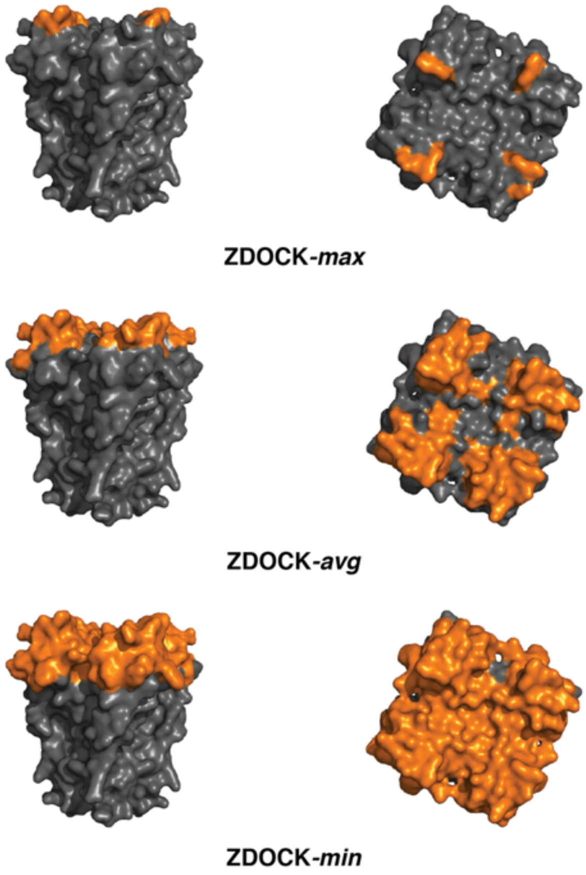

Fig. S2. Sidechain i-RMSD (**A**) and FNAT (**B**) comparison of all models (backbone) i-RMSD < 6Å before (y-axis) and after (x-axis) refinement (183 in total). Source data are provided as a excel file.

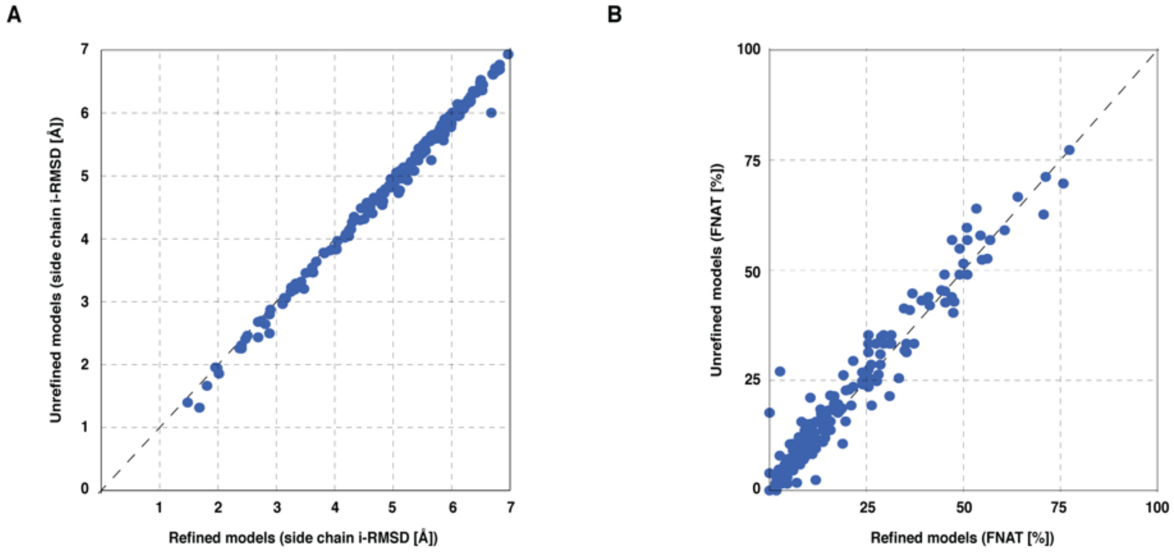

Fig. S3. ZDOCK success rate for the different tested (pseudo)membrane scenarios in the full dataset (18 cases). Source data are provided as a excel file.

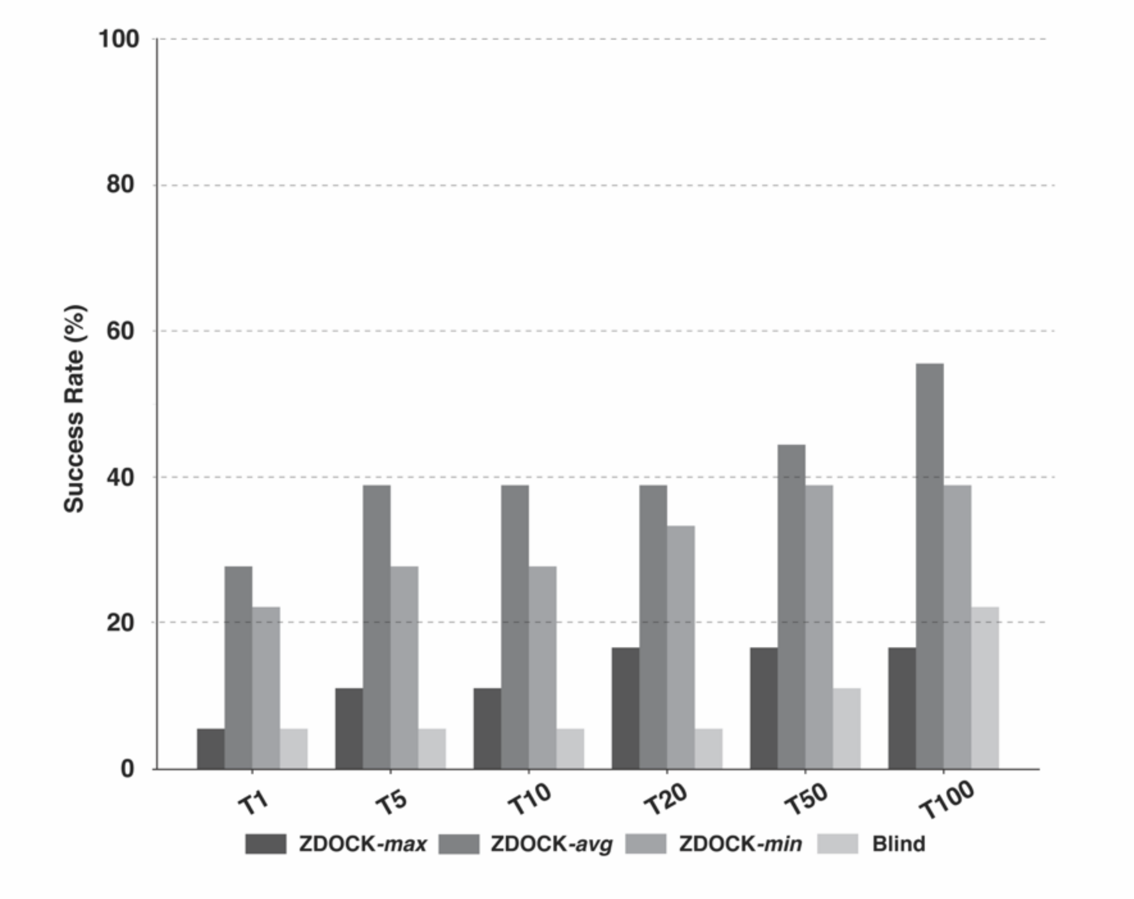

Fig. S4. Comparison of the membrane protein docking performance in the absence (Membrane) and presence (Membrane-*rst*) of experimental-like derived information (three defined interface residues (see Table S1) on the ligand (soluble) protein). The success rate is defined as the percentage of cases for which an acceptable or higher quality model was found within the selected top *N* models. These results include α-Helical and β-Barrel cases, 7 and 5, respectively. Source data are provided as a excel file.

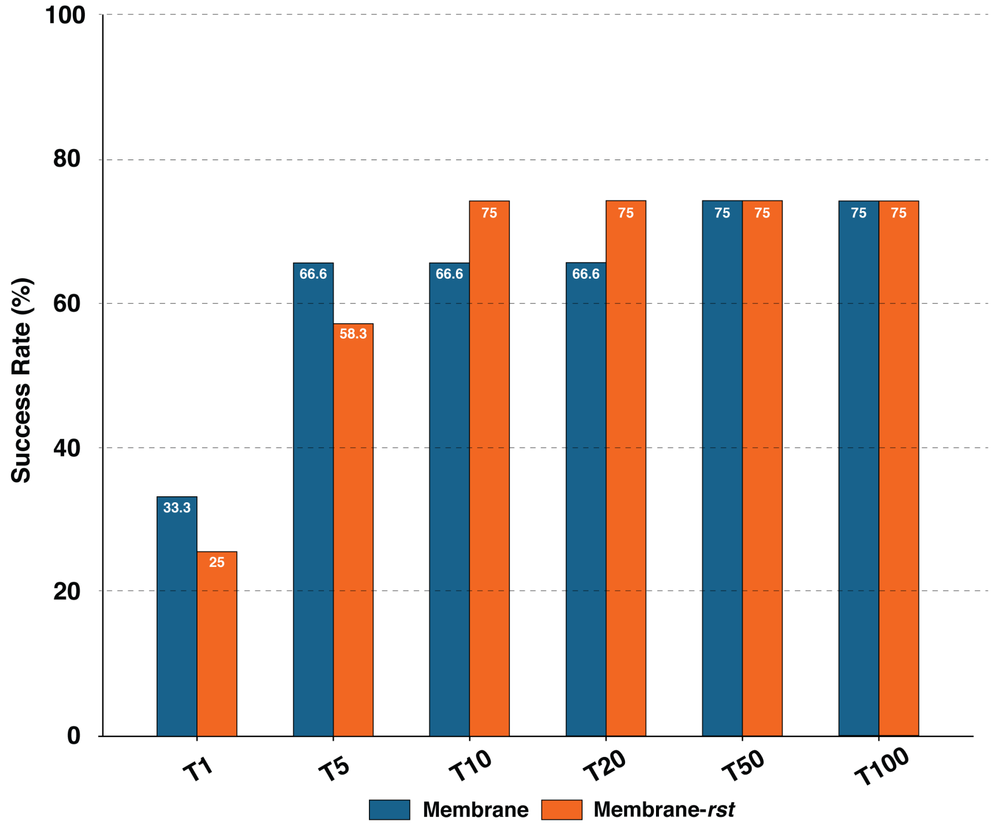

Table S1. List of the residues used in LightDock as active restraints during the docking process for each of the 12 tested cases.

Case	Residues
2bs2	ARG1071, ARG1167, ARG1232
2vpz	TYR1102, GLU1189, THR1072
4huq	GLN96, VAL377, GLU390
3x29	ARG227, ASN218, TYR310
2r6g-peri	GLN49, ASP207, GLN335
2r6g-cyto	LEU52, LYS132, HIS1089
2hi7	HIS32, ARG148, VAL150
2hdi	ASP311, ARG313, GLY357
2gsk	ARG204, LYS207, ARG212
3csl	ASN41, PHE78, ASP102
5d0o	ARG60, ARG135, ASP136
3v8x	LYS365, ASP416, LYS557
