# Supplementary material for: Integrative modeling of membrane-associated protein assemblies
Source: Nat Commun. 2020 Dec 4;11:6210. doi: 10.1038/s41467-020-20076-5 (PMC7718903; doi:10.1038/s41467-020-20076-5)
Supplement: Supplementary file 3 — Reporting Summary [file 41467_2020_20076_MOESM3_ESM.pdf]

nature research

Corresponding author(s): A.M.J.J. Bonvin and B. Jiménez-García

Last updated by author(s): Nov 10, 2020

Reporting Summary

Nature Research wishes to improve the reproducibility of the work that we publish. This form provides structure for consistency and transparency in reporting. For further information on Nature Research policies, see our Editorial Policies and the Editorial Policy Checklist.

Statistics

For all statistical analyses, confirm that the following items are present in the figure legend, table legend, main text, or Methods section.

n/a	Confirmed	
☐	☒	The exact sample size (*n*) for each experimental group/condition, given as a discrete number and unit of measurement
☒	☐	A statement on whether measurements were taken from distinct samples or whether the same sample was measured repeatedly
☒	☐	The statistical test(s) used AND whether they are one- or two-sided<br>*Only common tests should be described solely by name; describe more complex techniques in the Methods section.*
☒	☐	A description of all covariates tested
☒	☐	A description of any assumptions or corrections, such as tests of normality and adjustment for multiple comparisons
☒	☐	A full description of the statistical parameters including central tendency (e.g. means) or other basic estimates (e.g. regression coefficient) AND variation (e.g. standard deviation) or associated estimates of uncertainty (e.g. confidence intervals)
☒	☐	For null hypothesis testing, the test statistic (e.g. *F*, *t*, *r*) with confidence intervals, effect sizes, degrees of freedom and *P* value noted<br>*Give P values as exact values whenever suitable.*
☒	☐	For Bayesian analysis, information on the choice of priors and Markov chain Monte Carlo settings
☒	☐	For hierarchical and complex designs, identification of the appropriate level for tests and full reporting of outcomes
☒	☐	Estimates of effect sizes (e.g. Cohen's *d*, Pearson's *r*), indicating how they were calculated

Our web collection on statistics for biologists contains articles on many of the points above.

Software and code

Policy information about availability of computer code

Data collection	Docking tools:<br>- LightDock version 0.8.0 (Python package: https://pypi.org/project/lightdock/0.8.0/, Source Code: https://github.com/lightdock/lightdock/releases/tag/0.8.0).<br>- ZDOCK version 3.0.2 (http://zdock.umassmed.edu/software/)<br><br>Refinement tools:<br>- HADDOCK version 2.4 (https://www.bonvinlab.org/software/haddock2.4/download/).<br><br>A full demo on how to install the software and running the full protocol is available at: https://github.com/lightdock/membrane_docking/blob/master/demo/README.md
Data analysis	- PyMOL version 2.4.0 (https://pymol.org/2/)<br>- ProFit version 3.1 (http://www.bioinf.org.uk/software/profit/)<br>- Python version 3.8 (https://www.python.org/downloads/)

For manuscripts utilizing custom algorithms or software that are central to the research but not yet described in published literature, software must be made available to editors and reviewers. We strongly encourage code deposition in a community repository (e.g. GitHub). See the Nature Research guidelines for submitting code & software for further information.

Data

Policy information about availability of data

All manuscripts must include a data availability statement. This statement should provide the following information, where applicable:

- Accession codes, unique identifiers, or web links for publicly available datasets
- A list of figures that have associated raw data
- A description of any restrictions on data availability

Code to reproduce results presented in this manuscript as well as the membrane-associated protein docked/refined models can be found at https://github.com/lightdock/membrane_docking. The unbound structures from the MemCplxDB benchmark set tested in this manuscript can be found at https://github.com/haddocking/MemCplxDB. An online tutorial concerning structure preparation and docking with LightDock is available at https://lightdock.org/tutorials/membrane. Further reference and help on how to refine models with the new Haddock2.4 server can be found at http://www.bonvinlab.org/software/haddock2.4/tips/advanced_refinement/.

Field-specific reporting

Please select the one below that is the best fit for your research. If you are not sure, read the appropriate sections before making your selection.

☒ Life sciences ☐ Behavioural & social sciences ☐ Ecological, evolutionary & environmental sciences

For a reference copy of the document with all sections, see nature.com/documents/nr-reporting-summary-flat.pdf

Life sciences study design

All studies must disclose on these points even when the disclosure is negative.

Sample size	All complexes from the MemCplxDB (https://github.com/haddocking/MemCplxDB/) database whose interface lies between the membrane and either cytosolic or periplasmic regions were selected.
Data exclusions	No data were excluded from the analysis.
Replication	Running on different hardware might lead to small numeric differences. For this study, replication (same hardware) is achieved by using the same simulation seed in all replicas in LightDock. Two executions for every simulation have been performed with identical results.
Randomization	Randomization of the sample does not apply to the current study. In any case, starting poses are randomized (translation and rotation spaces) in LightDock as the protocol first step.
Blinding	This does not apply to the current study as the success rate of predictions is addressed using a public benchmark (MemCplxDB, https://github.com/haddocking/MemCplxDB)

Reporting for specific materials, systems and methods

We require information from authors about some types of materials, experimental systems and methods used in many studies. Here, indicate whether each material, system or method listed is relevant to your study. If you are not sure if a list item applies to your research, read the appropriate section before selecting a response.

Materials & experimental systems

n/a	Involved in the study
☒	☐ Antibodies
☒	☐ Eukaryotic cell lines
☒	☐ Palaeontology and archaeology
☒	☐ Animals and other organisms
☒	☐ Human research participants
☒	☐ Clinical data
☒	☐ Dual use research of concern

Methods

n/a	Involved in the study
☒	☐ ChIP-seq
☒	☐ Flow cytometry
☒	☐ MRI-based neuroimaging
